# Responding to the Article: A Qualitative Systematic Review of Healthcare Practitioners’ Experience of Workplace Violence

**DOI:** 10.21315/mjms-01-2025-054

**Published:** 2025-04-30

**Authors:** Erkan Boga

**Affiliations:** Esenyurt Necmi Kadıoğlu State Hospital, İstanbul, Turkey

Dear Editor,

I am writing to express my appreciation for the article titled “A Qualitative Systematic Review of Healthcare Practitioners’ Experience of Workplace Violence” (1), recently published in the *Malaysian Journal of Medical Sciences*. This systematic review addresses an issue of paramount importance and highlights the multifaceted nature of workplace violence in healthcare settings.

The review emphasises verbal violence as the most common form of aggression faced by healthcare practitioners. This observation aligns with findings in various global contexts, where verbal abuse, often characterised by humiliation or derogatory language, contributes significantly to emotional distress and reduced job satisfaction among healthcare administrators and professionals (2). The identification of factors such as miscommunication, long waiting times, and unrealistic patient expectations further underscores the systemic challenges that exacerbate these incidents. Addressing these root causes should be a priority for healthcare.

Another critical insight is the underreporting of cases, as highlighted in the article. Studies have shown that healthcare workers often refrain from reporting incidents due to a lack of institutional support or fear of retaliation (3). Creating an environment in which practitioners feel safe and supported is essential for reducing the prevalence of workplace violence. Policies that encourage transparent reporting and provide adequate follow-up are necessary steps towards achieving this goal.

The article also points out the limited number of qualitative studies conducted in Malaysia on this topic. This gap in research limits our understanding of how cultural and institutional factors influence the dynamics of workplace violence locally. For example, in Malaysia, the hierarchical nature of healthcare institutions and specific cultural sensitivities may affect how violence is perceived and addressed. Localised studies could provide invaluable insights into these nuances and inform the development of targeted interventions (4). In conclusion, this article not only highlights the pervasive issue of workplace violence in healthcare but also provides actionable recommendations for mitigating its impact. Strengthening institutional policies, enhancing communication training, and fostering a culture of safety and respect are vital steps forward. Future research should prioritise local contexts to bridge existing gaps in knowledge and develop effective, culturally sensitive solutions.

Thank you for addressing this critical topic and providing a platform for discussion.
